# Spatiotemporal Regulation of Ligand Trafficking and TLR9 Activation by PIEZO1 in Human Plasmacytoid Dendritic Cells

**DOI:** 10.34133/research.1067

**Published:** 2026-01-22

**Authors:** Shrestha Pattanayak, Deblina Raychaudhuri, Purbita Bandopadhyay, Upasana Mukhopadhyay, Tithi Mandal, Chinky Shiu Chen Liu, Nidhi Kalidas, Sumangal Roychowdhury, Krishnananda Chattopadhyay, Fnu Ashish, Bidisha Sinha, Dipyaman Ganguly

**Affiliations:** ^1^ IICB-Translational Research Unit of Excellence, CSIR-Indian Institute of Chemical Biology, Kolkata, India.; ^2^ Department of Biology, Trivedi School of Biosciences, Ashoka University, Sonipat, India.; ^3^ Department of Biological Sciences, Indian Institute of Science Education and Research, Kolkata, India.; ^4^ CSIR-Institute of Microbial Technology, Chandigarh, India.; ^5^ Structural Biology and Bioinformatics Division, CSIR-Indian Institute of Chemical Biology, Kolkata, India.; ^6^ Datla Human Immunome Lab, Trivedi School of Biosciences, Ashoka University, Sonipat, India.

## Abstract

Plasmacytoid dendritic cells (pDCs) are innate immune cells that produce type I interferons (IFNs) upon sensing nucleic acids via Toll-like receptor 9 (TLR9). Synthetic oligodeoxynucleotides CpGA and CpGB are widely used TLR9 agonists, yet only CpGA robustly induces IFN-α in pDCs. In contrast, CpGB drives much less IFN production. The mechanism underlying this ligand-specific response is not known. Here, we identify PIEZO1, a mechanosensory ion channel, as a regulator for this ligand-specific response. We show that CpGA, unlike CpGB, self-associates into large aggregates that generate membrane tension during cellular uptake, activating PIEZO1. This triggers calcium influx and localized F-actin assembly, retaining CpGA in early endosomes to sustain IRF7 activation and IFN production. PIEZO1 deficiency or inhibition abolishes CpGA-induced IFN responses, while PIEZO1 activation enhances IFN production by CpGB. Our findings reveal a hitherto unknown biophysical checkpoint in TLR9 signaling, where PIEZO1 translates membrane tension into spatially controlled TLR9 signaling. This study uncovers a novel role for mechanosensing in nucleic acid immunity, with implications for modulating IFN responses in infections and autoimmunity.

## Introduction

Plasmacytoid dendritic cells (pDCs) are a subset of innate immune cells specialized for the rapid and abundant production of type I interferons (IFNs) in response to nucleic acids [1,2]. This capacity is predominantly mediated through the endosomal recognition of unmethylated CpG motifs in DNA by Toll-like receptor 9 (TLR9) [3,4]. Synthetic oligodeoxynucleotides (ODNs) bearing CpG motifs have been widely employed to dissect pDC responses and are broadly classified into 3 categories—CpGA, CpGB, and CpGC, based on their structural modifications and immunostimulatory profiles [5]. Among these, CpGA is particularly potent at inducing type I IFNs, whereas CpGB predominantly triggers proinflammatory cytokine [tumor necrosis factor-α (TNF-α) and interleukin-6 (IL-6)] production but elicits a markedly blunted type I IFN response [5–7].

Thus, despite comparable binding affinities for TLR9, CpGA and CpGB elicit fundamentally distinct signaling outcomes, raising a longstanding and unresolved question: How do chemically related ligands engaging the same receptor drive divergent transcriptional programs in pDCs? Existing literature suggests that endosomal trafficking plays a central role in this dichotomy. CpGA is retained in early endosomes, where they interact with TLR9 driving IRF7 activation and type I IFN production, while CpGB is trafficked more rapidly to late endolysosomes, where it predominantly activates nuclear factor κB (NF-κB) [8,9]. This differential response based on ligand characteristic is interesting, more so because of inherent property of CpGA to form multimeric aggregates as opposed to CpGB [7,9]. Similar endolysosomal sorting has also been documented with immune complexes generated in the context of autoimmune diseases [10–12]. However, the upstream mechanisms that regulate ligand sorting and differential spatial compartmentalization of TLR9 signaling to induce type I IFN production remain poorly defined.

To address this, we initially investigated whether CpGA and CpGB differ in their modes of cellular entry or early endocytic uptake pathways in pDCs. Surprisingly, we did not find any marked differences in uptake efficiency or route of endocytosis between the 2 ligands. However, scanning electron microscopy revealed a striking difference: CpGA stimulation, unlike CpGB, resulted in pronounced membrane ruffling. Plasma membrane ruffling following internalization of bacteria has been demonstrated to induce membrane tension in local plasma membrane, which is sensed by PIEZO1 mechanosensors [13]. Thus, the membrane ruffling observed in response to CpGA uptake in human pDCs made us hypothesize a similar possibility.

We considered whether differences in the biophysical properties of the ligands, such as aggregation and cargo size, might be sensed by pDCs during uptake and contribute to their distinct functional outcomes. Specifically, we hypothesized that CpGA and CpGB may differentially engage cellular mechanotransduction pathways during internalization, thereby directing distinct intracellular trafficking and signaling outcomes. The mechanosensitive ion channel PIEZO1 has emerged as key sensors of membrane deformation and tension, regulating diverse biological processes ranging from vascular development to epithelial cell integrity [14]. Our previous work delineated the critical role of PIEZO1 in regulating human T cell activation, function, and migration [15–17]. However, whether mechanotransduction via PIEZO1 plays any role in innate immune sensing of nucleic acids and type I IFN response remains entirely unexplored.

Here, we identify PIEZO1 as a key mechanosensory gatekeeper that enables human pDCs to discriminate between CpG ligands based on their physical properties. We demonstrate that CpGA, but not CpGB, forms large self-associated molecular aggregates that result in greater increase in plasma membrane tension during cellular entry. This tension is sensed by PIEZO1, which becomes rapidly activated and clustered at sites of CpGA uptake, leading to localized calcium influx. In turn, PIEZO1-mediated calcium signaling drives filamentous actin (F-actin) polymerization around early endosomes, forming a stabilizing meshwork that retains CpGA in these signaling-permissive compartments and sustains IRF7 activation and nuclear translocation. Genetic or pharmacological inhibition of PIEZO1 disrupts this axis, resulting in premature early endosomal escape of CpGA, impaired IRF7 nuclear translocation, and a sharp reduction in IFN-α production. Strikingly, chemical activation of PIEZO1 is sufficient to enhance IFN production in response to CpGB, highlighting a ligand-independent mechanism to tune type I IFN responses in pDCs.

Collectively, our findings reveal that PIEZO1-mediated mechanosensation serves as a critical upstream regulator of CpGA trafficking and type I IFN production in pDCs. This study provides the first evidence that innate immune recognition of nucleic acids is determined not only by ligand chemistry or receptor specificity but also by the mechanical forces generated during antigen encounter. By coupling membrane tension to peri-endosomal actin remodeling and transcriptional output, PIEZO1 enables pDCs to convert physical properties of nucleic acid ligands into spatially and temporally precise cytokine responses. Thus, these insights not only address the long-standing question of CpG class-specific signaling but also perhaps highlight a more general principle by which innate immune cells interpret the physical properties of ligands to fine-tune cytokine output. Overall, the findings from this study not only resolve a key mechanistic gap in our understanding of TLR9 activation in human pDCs but also indicate possible new directions to modulate pDC activation and type I IFN-driven antiviral immunity and autoimmunity through the biophysical tuning of the structural attributes of TLR ligands or combination therapies targeting the PIEZO1 mechanosensors.

## Results

### Self-association prone CpGA ligands generate greater membrane tension than CpGB during internalization by primary human pDCs

**Fig. 1.**
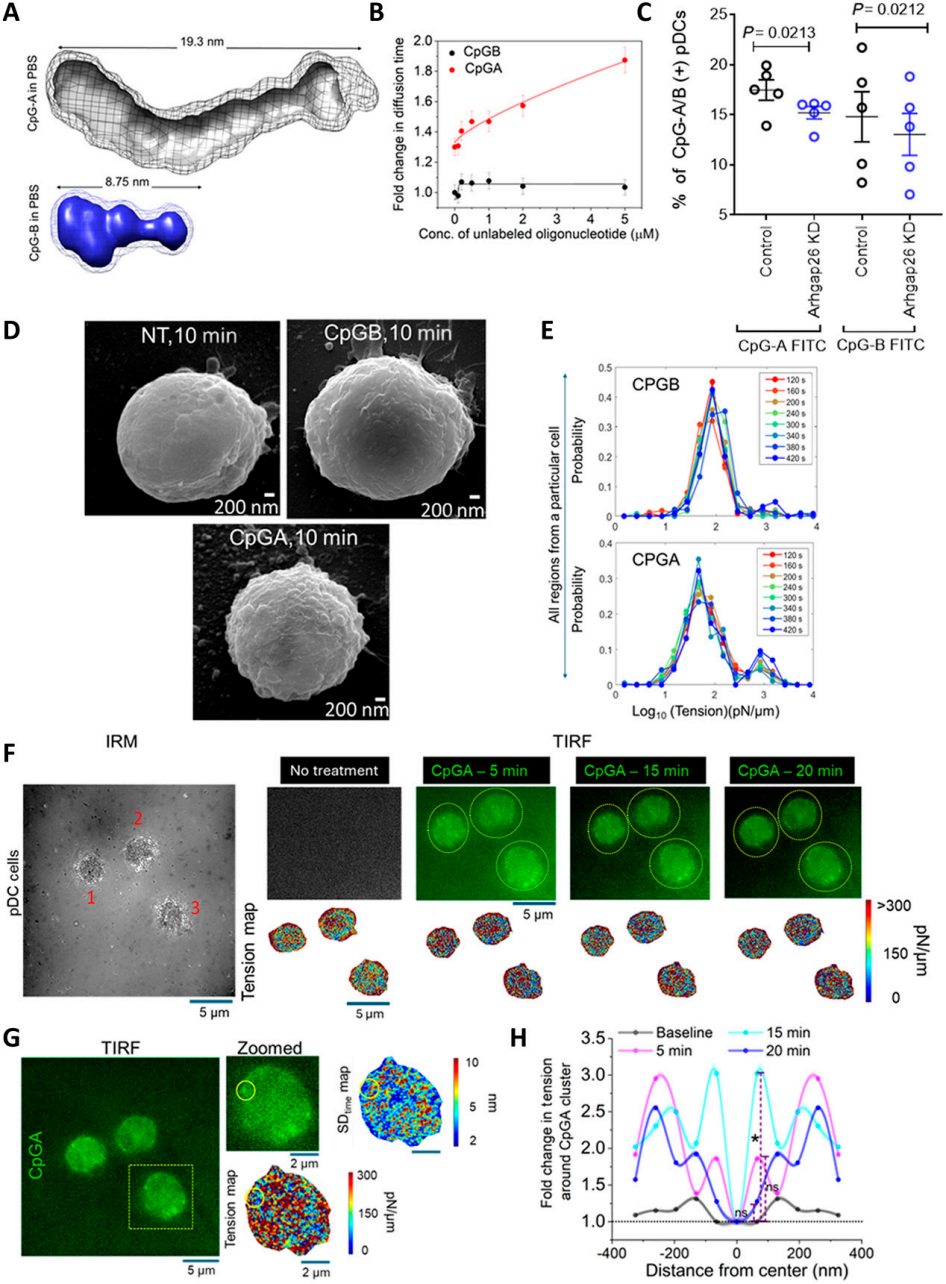
Self-associated CpGA aggregates generate greater membrane tension than CpGB during internalization by primary human pDCs. (A) Molecular maps of the envelope shapes, calculated for CpGA (gray) and CpGB (blue) molecules in PBS, are presented. Solid surface represents the common shape in 10 restorations, and mesh around represents the variation in the individual models computed. Numbers mentioned are measured approximate end-to-end dimensions in nanometers and correlate with persistence length calculated from respective SAXS datasets. (B) Plot of fold change in the diffusion time (with respect to 20 nM Alexa Fluor 488-labeled CpGB) versus concentration of unlabeled oligonucleotide (CpGA in red and CpGB in black) observed in FCS experiment. CpGB did not show any significant change in the diffusion time over the indicated range, whereas CpGA showed a change in diffusion time indicating self-association. Three independent measurements were taken (*n* = 3). The data were plotted as mean ± SD. (C) Flow cytometric analysis of Alexa Fluor 488-labeled CpGA or CpGB uptake in control and ARHGAP26 knockdown pDCs (*n* = 5). Data represent mean ± SEM from 3 independent experiments. Statistical significance was assessed using Student’s *t* test. (D) Representative scanning electron microscopy images demonstrating membrane morphology in untreated pDCs, or cells stimulated with CpGA or CpGB for 10 min. Images are representative of 4 independent experiments. (E) Evolution of tension distribution for different regions of a particular cell for 2 min followed in time after treatment with CpGB/A. (F) Representative IRM image of cells followed in time before and after CpGA-FITC addition, with their corresponding TIRF fluorescence images attached. The pixel-wise tension maps of the cells along time are also represented. Scale bar, 5 μm. (G) Typical TIRF frame with cells treated with CpGA-FITC. Scale bar, 5 μm. The dotted yellow rectangular line marks the cell that is zoomed in. The zoomed-in view shows a CpGA cluster with its corresponding pixel-wise tension and SD_time_ maps. Scale bar, 2 μm. The yellow circle marks the cluster. (H) Radial profiles of tension around the fluorescence peaks of CpGA clusters. Error denoted by the shaded region as SEM. *Significant change from the baseline condition for that particular time point after CpGA addition within 100 nm from center. Mann–Whitney *U* statistical significance test: ns, *P* > 0.05, *P < 0.05, **P < 0.001. The dotted black line through the plots denotes the value “1”. For CpGA, Ncell: 9; Ncluster: 46.

To investigate how the physical and structural properties of synthetic TLR9 ligands influence their uptake and downstream signaling in pDCs, we first compared CpGA and CpGB in terms of their supramolecular organization in solution. Analyzing small-angle x-ray scattering (SAXS) and fluorescence correlation spectroscopy (FCS) data, we found that CpGA spontaneously forms high-molecular-weight aggregates in phosphate-buffered saline (PBS), whereas CpGB remains predominantly monomeric or assembles into only small oligomers (Fig. 1A and B), consistent with previous reports [7,9]. In their SAXS profiles, the downward trend of data points as *s* values approached 0 nm^−1^ confirmed that both CpGA and CpGB molecules adopt pronounced interparticulate effect in water (Fig. S1A and B). This phenomenon is known for charged molecules as they repel each other in solution, causing them to appear smaller than their usual hydrodynamic profile [18–20]. This effect occurs mainly in the absence of counter ions, and it disappeared completely when the samples were moved to PBS. Interestingly, the increment in temperature did not change the solution scattering profile of the molecules in either solvent, indicating that the solution shape of both CpGA and CpGB is stable in the solvents, with or without counter ions. Presuming globular shape profile, the Guinier approximation of the averaged datasets provided *R*_g_ values of 5.63 and 2.65 nm for CpGA and CpGB in PBS, respectively. Comparison with their values in water suggested that in PBS, CpGA molecules self-associate significantly, while CpGB molecules opens up slightly. Guinier analysis for rod-like shape provided *R*_c_ values of 0.78 and 0.80 nm for CpGA and CpGB in PBS, respectively. Deduced parameters provided *L* and *A* values of 19.31 and 8.75 nm, and 7.22 and 3.31 for CpGA and CpGB in PBS, respectively. This clearly indicated differential behavior of solution shape and self-association order for CpGA molecules, which appeared to now form exceptionally elongated shape with very skewed aspect ratio compared to CpGB molecules. Dimensionless Kratky plots for both molecules peaked close to s*R*_g_ value of 1.73 (dotted lines in middle panels of Fig. S1A and B), which suggested disordered globular shape [21,22]. Bayesian inference of the SAXS datasets indicated average molecular weight of 53.1 and 9.5 kDa for CpGA and CpGB molecules in PBS. Molecular envelope shapes were calculated for both CpGA and CpGB molecules in PBS and water (Fig. 1A and Fig. S1C). Notably, the minimal aggregate size of CpGA was more than twice that of CpGB in PBS, demonstrating substantial differences in particle size and structural complexity between the 2 ligands (Fig. 1A). However, there was no difference in the size of CpGA and CpGB in water (Fig. S1C).

Next, to assess the self-association propensity between CpGA and CpGB oligonucleotide, we used single-molecule fluorescence-based FCS. FCS measures the fluctuation of fluorescence intensity in a small detection volume (~fl) to determine the dynamics of the biomolecules [23]. We used a concentration-dependent study by using 20 nM Alexa Fluor 488-labeled oligonucleotide (CpGA or CpGB) with increasing concentration of unlabeled oligonucleotide (CpGA or CpGB) up to 5 μM. In the case of CpGB, we did not observe any significant shift in the normalized autocorrelation function (Fig. S1D) and in the fold change in the diffusion time obtained from FCS measurements (Fig. 1B). Any change in the number of particle (*N*) present in the confocal volume indicates association or dissociation of the detected species. A decrease in the number of particles inside the detection volume indicates self-association or oligomerization of the species under consideration [24,25]. The number of particle (*N*) analysis from FCS curve fitting using 3-dimensional (3D) Gaussian single diffusing species did not show any significant change for the entire concentration change (Fig. S1F). All together, these confirmed the absence of any higher-order species formed by CpGB in the solution. On the contrary, for CpGA, we observed a shift in the normalized autocorrelation function (Fig. S1E) and also a significant change of the fold change in the diffusion time (Fig. 1B). With 1 μM unlabeled CpGA concentration, the number of monomers associated is ~3 (assuming a cube root dependence of diffusion with molecular weight), and with 5 μM unlabeled CpGA concentration, the number of monomers associated is ~6. The number of particle (*N*) analysis for CpGA showed a significant decrease indicating self-association (Fig. S1F).

Engagement of different endocytic pathways (viz. clathrin-mediated versus caveolin-mediated) has been shown to be regulated by the size of the cargo as shown earlier using different sized microspheres [26]. This raised a critical question: Do these differences in aggregation state and particle size contribute to their divergent functional outcomes in pDCs by engaging distinct endocytic pathways? To address this, we used pharmacological inhibitors targeting clathrin- and caveolin-mediated endocytosis—Pitstop 2 and Genistein, respectively [27,28]. Inhibition of these classical uptake routes did not significantly impair internalization of either CpGA or CpGB (Fig. S2A), indicating that both ligands are likely internalized through noncanonical pathways. We next focused on the clathrin- and caveolin-independent [clathrin-independent carriers/glycosylphosphotidylinositol-anchored protein (GPI-AP)-enriched compartments] pathway and found that small interfering RNA (siRNA)-mediated knockdown of ARHGAP26 (Fig. S2B), the driver adaptor molecule of CLIC-GEEC endocytosis [29], led to reduced uptake of both CpGA and CpGB (Fig. 1C and Fig. S2C). These findings indicated that the CLIC-GEEC pathway is involved in the internalization of both ligands and that differences in their functional outcomes are unlikely to be driven by differential endocytic routing. Interestingly, scanning electron microscopy of primary human pDCs treated with CpGA or CpGB for 10 min demonstrated striking differences in membrane morphology. CpGA-treated cells exhibited markedly more membrane ruffling compared to CpGB-treated cells (Fig. 1D). This observation led us to hypothesize that the internalization of the larger CpGA cargo leads to greater membrane deformation, thereby imposing greater membrane stretch and mechanical strain on the plasma membrane, potentially serving as a mechanical cue.

### Increased local membrane tension surrounding endocytic pits during uptake of CpGA by human pDCs

To determine whether the larger size of self-associated aggregates of CpGA induces a global increase in the basal cell membrane tension during uptake, we performed interference reflection microscopy (IRM) on cells before and after administration of CpGs. IRM is a noninvasive live cell imaging technique to quantify the nanometric height fluctuations of the basal membrane of adherent cells [30]. The characteristics of the height fluctuations of the membrane are primarily determined by its mechanical state. Hence, by analyzing spatiotemporal patterns of the height fluctuations, especially by fitting the power spectral density (PSD) with Helfrich-based model, estimates of the mechanical parameters, including fluctuation tension [31]—the effective tension of the basal membrane, could be derived as previously reported [30]. On following same cells after addition of CpGA/B (Movie S1), we observed that CpGA, but not CpGB, increased the probability of finding higher tensed membrane patches in the cells. This is symbolized by the second peak of probability at higher tension values visible for CpGA but not for CpGB (Fig. S2D). The amplitude of the height fluctuations was quantified by measuring the standard deviation of relative height from time series of height fluctuations (termed SD_time_). There was a concomitant pronounced decrease in SD_time_ as tension increased (Fig. S2E). Further, following single cells through time clearly revealed the building of the second peak in tension with time (Fig. 1E), indicating that certain regions within CpGA-treated cells had enhanced tension rather than only the average tension being increased.

Therefore, we next focused on understanding the impact on the membrane close to the cluster of CpGA. We utilized a sequential correlative imaging approach combining total internal reflection fluorescence (TIRF) microscopy and IRM (Fig. S3A) and imaged cells over time (Fig. S3B). TIRF enabled imaging of fluorescently labeled CpGA close to the basal membrane, due to low penetration depth of the evanescent mode, while IRM enabled mapping of the effective tension (Fig. 1F) and SD_time_ (Fig. S3C) in these single cells over time. Particular clusters were identified (Fig. 1G) through TIRF images and maps of tension and SD_time_ generated from IRM images. To quantify the local effect, line scans of the tension, SD_time_, and fluorescence profiles were performed along *x* and *y* directions, centered at fluorescence peaks (indicating center of the cluster) (Fig. S3D). The averaged normalized profiles of tension around the fluorescence peak depict the fold change in tension as one moved away from the center. Such profiles were plotted for the 5-, 15-, and 20-min time points (Fig. 1H) along with profiles of the fluorescence at those regions (Fig. S3E). The baseline (or untreated) (Fig. 1H and Fig. S4A) represents the state of tension at the same region where CpGA/B later clusters after its addition. By 15 min, we observed a distinct increase in membrane tension within approximately 100 nm of the CpGA cargo compared to the central region (Fig. 1H), which is not observed in case of CpGB treatment at the cluster fluorescence center (Fig. S4B and C). Although membrane tension here is dependent on the form of Helfrich model chosen to fit the fluctuation spectra, we demonstrate that direct measurements of membrane fluctuation amplitudes also fully reflect the impact of local membrane remodeling by CpGA, as the increase in effective tension was accompanied by a decrease in SD_time_ (Fig. S3E). This supports the idea that CpGA (Fig. S3E) uptake, but not CpGB (Fig. S4D), locally elevates membrane tension around its site, thereby decreasing SD_time_. From the cluster center, the maximum surge in fluctuation tension produced by CpGA cargo, within 325 nm, was compared with the untreated condition. We find the maximum tension increase (difference of tension at pixels from tension at center of CpGA cluster) to be lower when untreated while peaking at ~1,300 pN/μm at 5 and 15 min after CpGA addition (Fig. S3F). Interestingly, Piezo activation has been known to be triggered at a tension range of ~600 to 2,100 pN/μm [32–37], although the tension threshold is expected to be system-dependent. The tension increase by CpGA, measured in this data (Fig. S3F) at 15 min in comparison to baseline (Fig. S3G), thus, could be sufficient to trigger PIEZO1 activation, which remains statistically insignificant in case of CpGB treatment (Fig. S4E and F).

Overall, these findings suggested that the differential mechanical impact of CpGA versus CpGB is a function of the cargo size and resulting membrane tension, rather than receptor-specific endocytic sorting pathways.

### CpGA-induced membrane tension activates PIEZO1 to selectively enhance IFN-α production by human pDCs

**Fig. 2.**
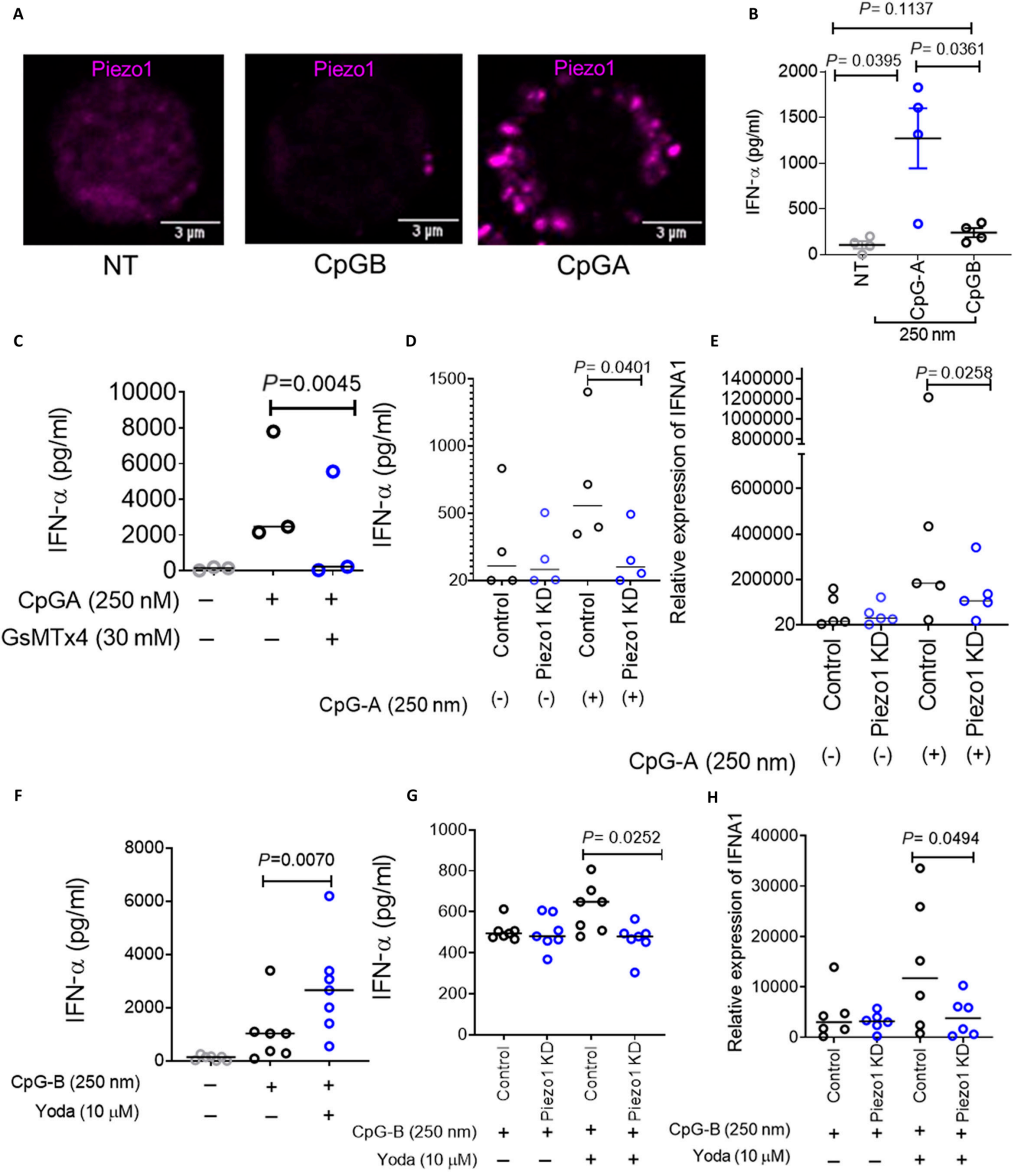
CpGA-induced membrane tension activates PIEZO1 to selectively enhance IFN-α production by pDCs. (A) Representative confocal microscopy images showing intracellular PIEZO1 distribution in untreated pDCs and those treated with CpGA or CpGB. (B and C) IFN-α production in pDC culture supernatants following stimulation with CpGA or CpGB (B), and CpGA with increasing doses of GsMTx4 (C), measured by ELISA. *n* = 3 to 4. (D and E) IFN-α secretion (D) and *IFNA1* mRNA expression (E) in control and PIEZO1-knockdown (PIEZO1 KD) pDCs treated with or without CpGA. *n* = 4 to 7. (F) IFN-α production by pDCs stimulated with CpGB in the presence or absence of Yoda1. *n* = 7. (G and H) IFN-α secretion (G) and *IFNA1* gene expression (H) in control and PIEZO1 KD pDCs stimulated with CpGB with or without Yoda1 were measured. *n* = 6 to 7. Data represent median. Statistical analysis was performed using Student’s *t* test.

Given the pronounced localized increase in membrane tension during CpGA exposure, we hypothesized that the mechanosensitive ion channel PIEZO1 may serve as a mechanosensor linking ligand size to immune signaling. Consistent with our hypothesis, confocal microscopy revealed rapid membrane recruitment and clustering of PIEZO1 in pDCs following CpGA, but not CpGB treatment (Fig. 2A), suggesting that only CpGA induces sufficient membrane tension to cause PIEZO1 clustering. Functionally, CpGA triggered significantly higher levels of IFN-α compared to CpGB (Fig. 2B), whereas CpGB induced higher production of TNF-α in primary human pDCs (Fig. S5A), consistent with previous reports describing distinct cytokine profiles for these ligands. Therefore, to assess whether PIEZO1 activation contributes to the CpGA-specific type I IFN response in pDCs, we quantified IFN-α secretion following PIEZO1 inhibition. Pharmacological inhibition with GsMTx4 [38,39] or siRNA-mediated knockdown of *PIEZO1* (Fig. S5B) significantly reduced CpGA-induced IFN-α levels at both the mRNA and protein levels (Fig. 2C to E). Further, to test whether PIEZO1 activation is essential to promote a CpGA-like IFN response, we costimulated CpGB-treated cells with Yoda1, a selective PIEZO1 agonist [40–42]. Yoda1 markedly enhanced CpGB-induced IFN-α production (Fig. 2F), and this increase was abrogated in PIEZO1 knockdown pDCs (Fig. 2G and H). These results demonstrated that PIEZO1 activation plays a critical role in driving type I IFN responses in pDCs. Together, these findings implicated PIEZO1 as a mechanosensor that directly links biophysical properties of TLR9 ligands to type I IFN production in human pDCs.

### PIEZO1 activation promotes CpGA retention in early endosomes and IRF7 nuclear translocation in human pDCs

**Fig. 3.**
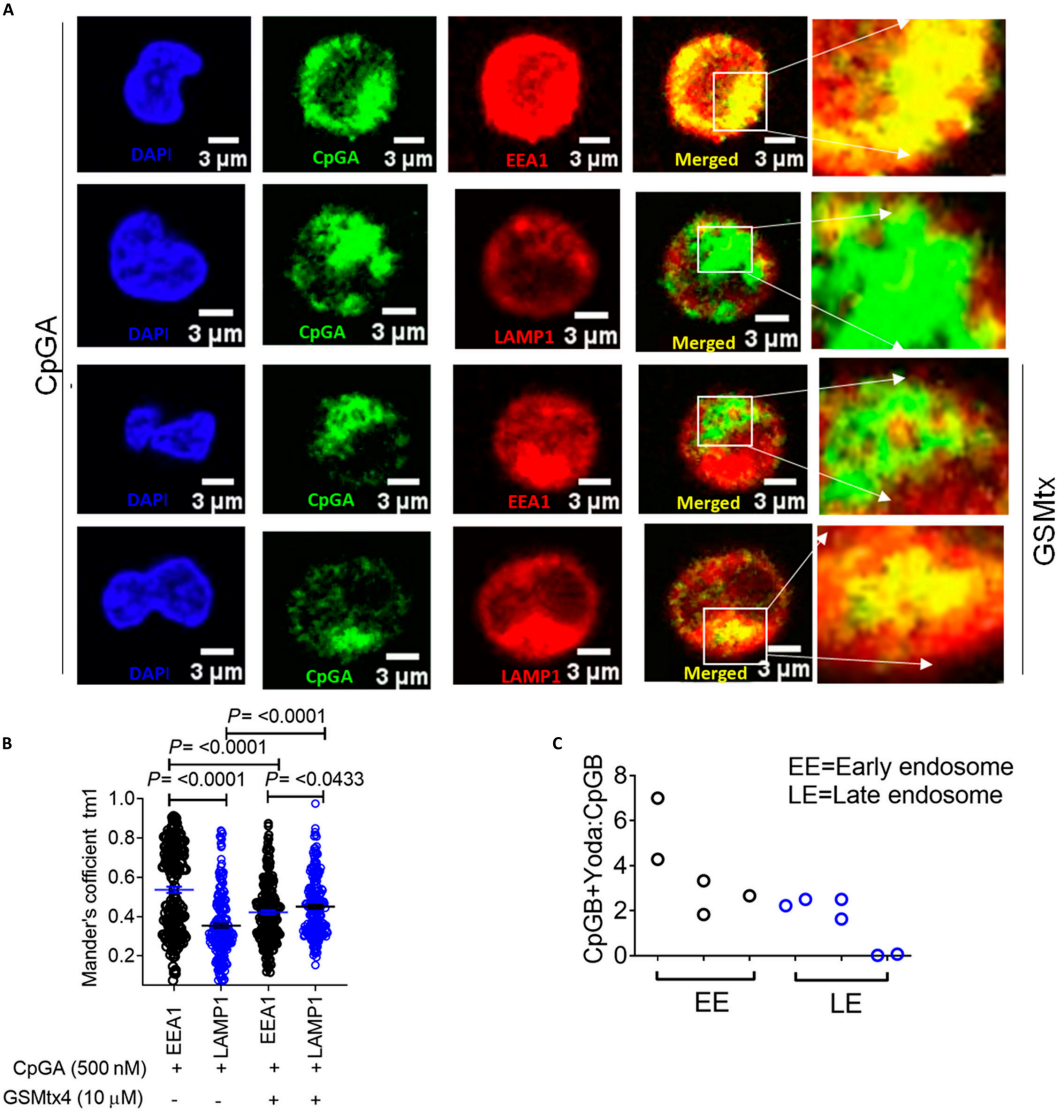
PIEZO1 activation promotes CpGA retention in early endosomes. (A) Representative confocal images of fixed, immunostained primary human pDCs following CpGA-FITC stimulation, visualizing early endosomes (EEA1) and late endosomes (LAMP1) under basal and GsMTx4-treated conditions. (B) Manders’ coefficient analysis quantifying colocalization of CpGA with EEA1 and LAMP1 under control and PIEZO1-inhibited (GsMTx4-treated) conditions (*n* > 195 cells per condition). (C) Dot plot showing relative detection [optical density (OD) 450 nm] of biotinylated CpGB in subcellular fractions of pDCs stimulated with CpGB-biotin ± Yoda1. Data represent mean ± SEM and are derived from 2 independent experiments.

We next investigated how PIEZO1 activation enhances type I IFN production by modulating the subcellular trafficking of CpGA. To determine the intracellular localization of CpGA, we performed confocal microscopy using markers for early endosomes (EEA1) and late endosomes (LAMP1). Consistent with previous studies, CpGA predominantly localized to early endosomes in primary human pDCs (Fig. 3A and B and Movies S2 and S3). Notably, this early endosomal retention of CpGA was dependent on PIEZO1 activity. Pharmacological inhibition of PIEZO1 using GsMTx4 significantly decreased CpGA colocalization with EEA1 and increased its colocalization with LAMP1 (Fig. 3A and B and Movies S4 and S5), suggesting a faster transition of CpGA to late endosomes and reduced residence time in early endosomes. Further, to confirm the critical role of PIEZO1 in regulating TLR9 ligand trafficking in pDCs, we cotreated CpGB-stimulated pDCs with Yoda1. Subcellular fractionation analysis showed that Yoda1 enhances CpGB accumulation in early endosomes (Fig. 3C), supporting the role of PIEZO1 in promoting retention of TLR9 ligands in early endosomal compartments, thereby prolonging their signaling potential.

**Fig. 4.**
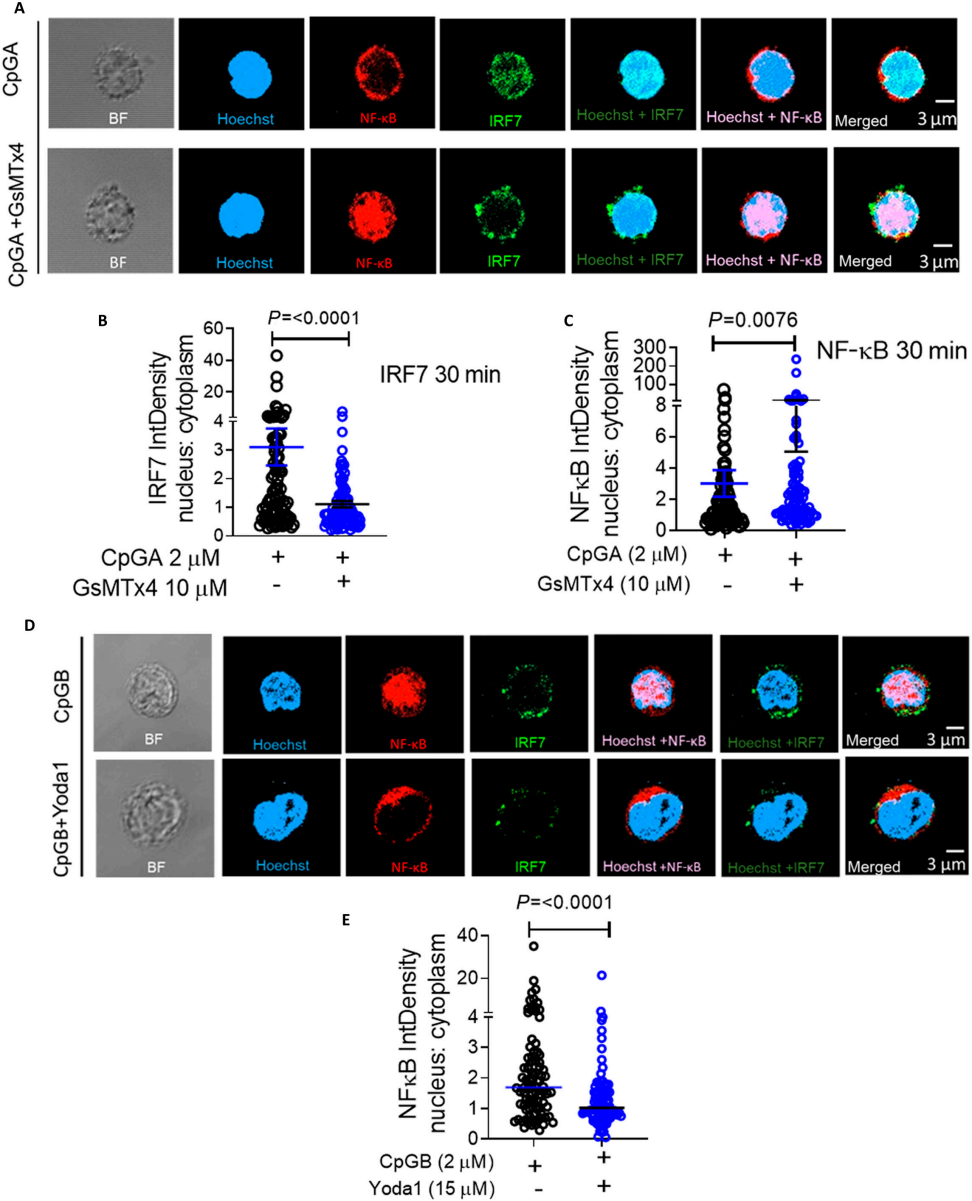
PIEZO1 activation favors nuclear translocation of IRF7 in response to TLR9 activation. (A) Representative confocal images of fixed, immunostained human primary pDCs showing subcellular localization (nuclear versus cytosolic) of NF-κB and IRF7 following CpGA stimulation under control and PIEZO1-inhibited conditions. (B and C) Quantitative analyses of nuclear-to-cytoplasmic (N:C) intensity ratios of IRF7 (B) and NF-κB (C) in CpGA-stimulated pDCs treated with or without GsMTx4. (D) Representative confocal images of subcellular localization of NF-κB in primary human pDCs treated with CpGB in the presence or absence of Yoda1. (E) Quantification of N:C intensity ratios of NF-κB in pDCs stimulated with CpGB in the presence or absence of Yoda1 (*n* > 90 cells per condition). All data are representative of at least 3 independent experiments. Student’s *t* test was used to calculate significance, and data are represented as mean ± SEM. Statistical analysis was performed using Student’s *t* test (B) or Mann–Whitney *U* test (E).

We next investigated the functional consequence of altered endosomal trafficking on downstream signaling and transcription factor localization. Nuclear localization studies using high-resolution imaging showed that CpGA retention in early endosomes is associated with robust translocation of IRF7 to the nucleus (Fig. 4A and Movie S6), a key step for initiating type I IFN gene transcription [8,43]. In contrast, inhibition of PIEZO1 significantly impaired nuclear localization of IRF7 following CpGA stimulation and led to increased nuclear translocation of NF-κB, consistent with enhanced CpGA routing to late endosomes (Fig. 4A to C and Movie S7). Further, as expected, we observed a reduction in total p-IRF7 levels in the presence of GsMTx4, consistent with reduced activation of the IFN-α induction pathway in treated pDCs (Fig. S5C). We also assessed whether PIEZO1 activation could alter CpGB-driven nuclear translocation of transcription factors. As expected, CpGB stimulation led to NF-κB translocation (Fig. 4D) into the nucleus. However, cotreatment with the PIEZO1 agonist Yoda1 attenuated NF-κB translocation to the nucleus, although significant nuclear translocation of IRF7 was not discernible at the same time point (Fig. 4D and E). Taken together, these results demonstrated that PIEZO1 plays a key role in controlling the endosomal trafficking of CpGA, prolonging its retention in early endosomes, thereby facilitating sustained IRF7 activation and IFN-α production in human pDCs.

### PIEZO1-driven calcium influx induces localized actin remodeling to stabilize CpGA-containing early endosomes in human pDCs

**Fig. 5.**
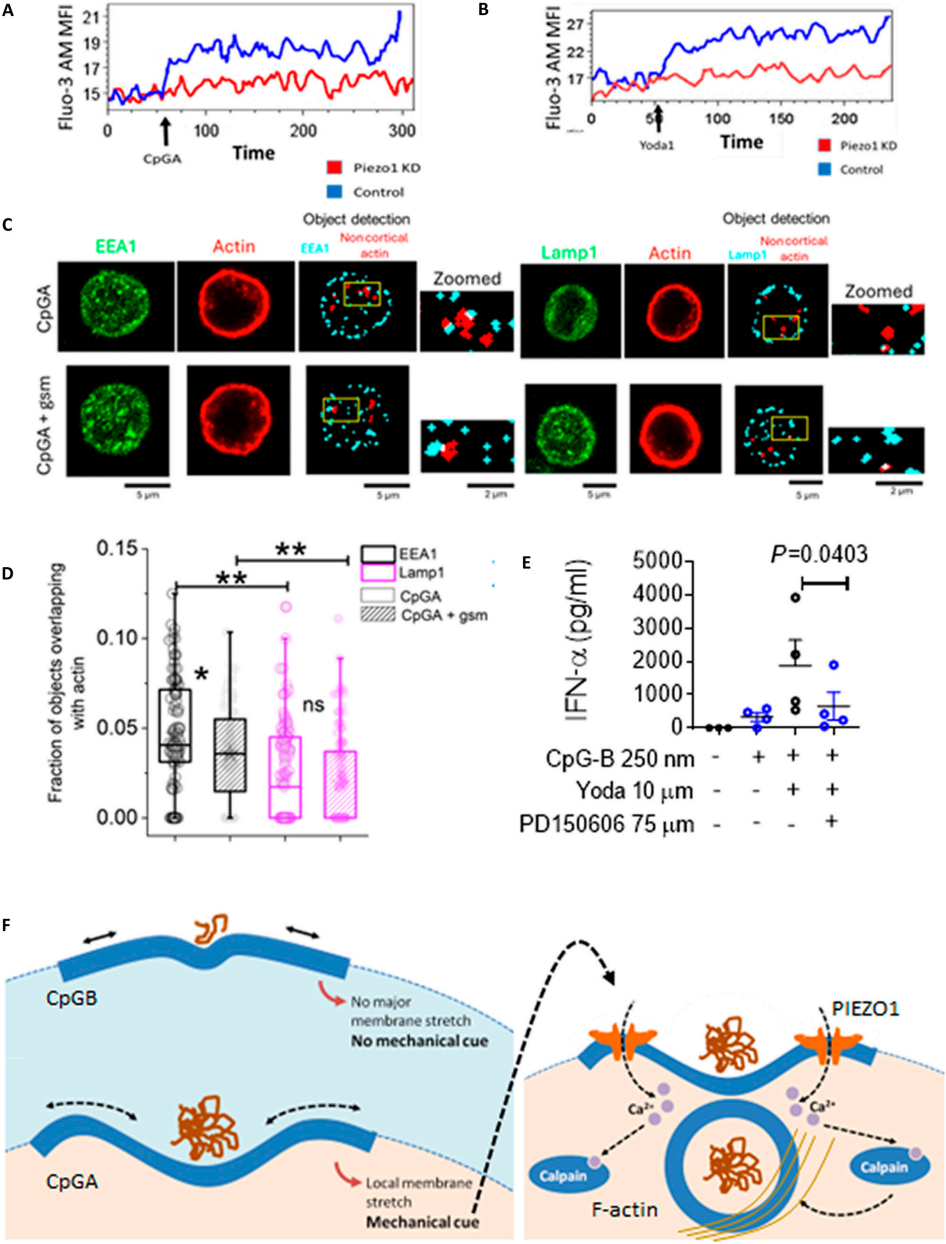
PIEZO1-driven calcium influx induces localized actin remodeling to stabilize CpGA-containing early endosomes. (A and B) Intracellular Ca^2+^ abundance in control and PIEZO1 KD pDCs treated with CpGA (A) or Yoda1 (B) was assessed by flow cytometry. Arrows represent the time point of addition of the indicated treatments. (C) Representative images of dual-channel confocal images of EEA1/LAMP1 in the green channel and actin labeled in red channel under CpGA and CpGA + GsMTx4-treated condition—along with the typical object detection (right side panel) observed away from the cortex (without cortical actin) for a particular z slice of a cell (insets shows the zoomed-in view of a few colocalized objects in each condition).The white pixel regions indicate the colocalized region of EEA1/LAMP1 with noncortical actin. xy-1 pixel = 0.13 μm, z step = 1 μm. (D) Cell-wise comparative study of the fraction of endosomal objects that overlap with actin under a particular treatment, with ** indicating a significant decrease in actin colocalization with early endosomes on PIEZO1 inhibition (without cortical actin). EEA1 (Ncell): CpGA, 116; CpGA + gsm, 116; LAMP1 (Ncell): CpGA, 136; CpGA + gsm, 108. Scale bar, 10 μm. Mann–Whitney *U* statistical significance test: ns, *P* > 0.05; *P < 0.05; **P < 0.001. (E) IFN-α production in supernatants of pDCs stimulated with CpGB in the presence of indicated treatments. *n* = 4. Student’s *t* test was performed. Data are representative of 2 independent experiments and represent mean ± SEM. (F) Schematic representing mechanism of differential activation of PIEZO1 by CpGA and CpGB as well as downstream mechanisms regulating PIEZO1-mediated CpGA retention in early endosomes.

We next investigated the molecular mechanisms by which PIEZO1 activation promotes CpGA retention in early endosomes. PIEZO1 is a calcium channel, and based on prior evidence from our group linking PIEZO1 channels to cytoskeletal regulation [15,17], we hypothesized that activation of PIEZO1 and local accumulation of Ca^2+^ may contribute to peripheral retention of the early endosomes through calcium-dependent local F-actin remodeling. To test this, first we measured intracellular calcium levels using a fluorescent calcium indicator (Fluo-3 AM) and flow cytometry. Both CpGA and Yoda1 induced a robust calcium influx in primary human pDCs, which was abolished following PIEZO1 knockdown (Fig. 5A and B), confirming PIEZO1 dependence. We then assessed whether PIEZO1-driven calcium signaling influences actin distribution, as documented earlier in the context of T cell activation and migration [15,17]. To examine the spatial organization of the F-actin network, we performed confocal microscopy of cells costained for F-actin and EEA1 or F-actin and LAMP1. Images were analyzed ensuring that the cortical F-actin (underlying the plasma membrane) was disregarded and only internal F-actin patches were considered (Fig. S6A and B). The data demonstrated higher F-actin enrichment around early endosomes (higher colocalization of actin-rich patches with EEA1-rich structures), compared to late endosomes (labeled with LAMP1) in CpGA-treated pDCs (Fig. 5C and D and Fig. S6A and B), suggesting preferential actin remodeling locally around the early endosomes. Importantly, this F-actin enrichment around early endosomes was significantly reduced upon PIEZO1 inhibition with GsMTx4, further supporting its dependence on PIEZO1-mediated signaling (Fig. 5C and D).

Next, to test whether F-actin remodeling is essential for PIEZO1-driven type I IFN production, we pharmacologically disrupted F-actin polymerization using the calpain inhibitor PD150606. This significantly reduced IFN-α production in cells costimulated with CpGB and Yoda1 (Fig. 5E). Cumulatively, these findings demonstrated that PIEZO1-dependent calcium influx initiates a localized actin remodeling program that stabilizes CpGA-containing early endosomes, thereby sustaining IRF7 activation and robust type I IFN responses in human pDCs (Fig. 5F).

## Discussion

The present study uncovers a previously unrecognized role for PIEZO1-mediated membrane tension sensing in regulating nucleic acid-induced type I IFN production in pDCs (Fig. 5F), revealing that innate immune responses are critically shaped not only by ligand chemistry or receptor specificity but also by the biophysical properties such as ligand’s propensity for self-association, increasing the cargo size and membrane tension. We demonstrate that in physiological condition, CpGA molecules, unlike CpGB, self-associate to form large aggregates that causes substantial membrane stretch during uptake. CpGA induces local membrane tension during cellular uptake, which activates PIEZO1 at the plasma membrane. This in turn triggers calcium influx and local actin polymerization leading to retention of CpGA within the early endosomal compartment for a longer duration, permitting sustained IRF7 activation and robust type I IFN production. Inhibition of PIEZO1 or downstream actin filament polymerization leads to premature CpGA trafficking toward late endosomes and abrogates IFN responses. Strikingly, chemical activation of PIEZO1 up-regulates IFN-α production in response to CpGB, underscoring the sufficiency of mechanotransduction in rewiring pDC signaling.

While much is known about pattern recognition receptor (PRR) specificity and endosomal trafficking in shaping innate immune signaling [44], this study introduces membrane tension and mechanosensing as critical upstream regulators of these pathways. The observation that CpGA-induced membrane deformation activates PIEZO1 to promote actin remodeling and endosomal retention positions PIEZO1 as a mechanosensory gatekeeper between ligand uptake and compartmentalized signaling. Notably, previous studies have described the importance of early endosomal localization for CpGA-mediated IRF7 activation [8,9], but the cues regulating this phenomenon remained elusive. Our findings provide a mechanistic link between physical properties of the ligand and subcellular signal routing, offering a new layer of regulation in the context of human pDC biology.

These insights also expand the current understanding of innate immunity beyond chemical recognition. Increasing evidence supports that physical attributes such as size, shape, and rigidity influence immune responses [45,46]. We now show that size of the CpGA cargo, through mechanical activation of PIEZO1, modulates TLR9 signaling. This may explain why CpG-based immunotherapies optimized solely for sequence or chemical stability have shown limited efficacy [47]. Our findings suggest that tuning the biophysical features of nucleic acid ligands could offer a new strategy to modulate IFN responses. While PIEZO1 has been implicated in other immune cell functions [15,17,48], this is the first report linking PIEZO1 to nucleic acid sensing and regulation of type I IFN induction by human pDCs.

Given the central role of pDCs and type I IFNs in autoimmune diseases such as lupus [12,49], our findings may hold particular relevance in these contexts. In autoimmune diseases, self-nucleic acids frequently form large complexes with the antimicrobial peptide LL37, which is an established danger molecule in different autoimmune contexts, viz. psoriasis and systemic lupus [10,11], or with autoantibodies [50,51]. These complexes also undergo aberrant endolysosomal sorting, leading to their accumulation triggering excessive type I IFN production, a key driver of inflammation and pathology [10,11]. Our findings can be extrapolated to suggest that such endogenous immune complexes carrying TLR ligands may lead to increase in local membrane tension during their uptake by pDCs, potentially activating PIEZO1 and modulating downstream type I IFN induction. These insights also identify PIEZO1 as a potential therapeutic target for modulating dysregulated IFN responses in the contexts of autoimmune diseases and autoreactive inflammation wherein a crucial role of pDC-derived type I IFNs is established [12].

The present study also raises compelling new questions. Is PIEZO1 a general modulator of nucleic acid sensing across other endosomal PRRs such as TLR7, TLR8, or TLR3? Might endogenous nucleic acid containing immunogenic complexes such as those observed in necrotic tissue or neutrophil extracellular traps exploit this pathway to drive autoimmune inflammation? These avenues of inquiry could extend the impact of our findings beyond CpG biology and into fundamental mechanisms of self-nonself discrimination and immune homeostasis.

Interestingly, a recent study also showed that chronic extracellular matrix stiffening in fibrotic skin suppresses TLR9-induced type I IFN production in pDCs via NRF2 activation, emphasizing the role of sustained mechanical cues in pDC regulation [52]. In contrast, our findings highlight that acute, ligand-intrinsic membrane tension driven by uptake of larger CpGA cargo activates Piezo1 to enhance IFN responses. These differing outcomes likely reflect distinct magnitudes, durations, and cellular/clinical contexts of mechanical input. Together, the findings suggest that pDCs integrate diverse mechanical signals to modulate type I IFN output in a context-dependent manner.

In summary, our work reveals that Piezo1 functions as a biophysical checkpoint that translates membrane tension into durable type I IFN signaling in pDCs, resolving a key mechanistic gap in TLR9 signaling biology and redefining how physical properties of ligands shape innate immune responses. By identifying mechanosensation as a central determinant of nucleic acid–induced immunity, we introduce a new conceptual axis in pDC regulation, one that integrates mechanical force, membrane dynamics, cytoskeletal remodeling, and transcriptional activation, and opens new therapeutic possibilities for harnessing or modulating IFN responses in health and disease with wide-ranging implications for autoimmunity, vaccine design, and immunotherapy.

## Methods

### Small-angle x-ray scattering

The SAXS datasets for studying the changes induced by solvent constitution on CpGA (ODN2216, InvivoGen) and CpGB (ODN2006, InvivoGen) were acquired on SAXSpace instrument (Anton Paar GmbH, Graz, Austria at CSIR-Institute of Microbial Technology, Chandigarh, India). Line collimation arising from a sealed x-ray tube was incident on the samples and buffer contained in a thermostated quartz capillary. For each dataset, one frame of 1 h of exposure to x-rays was considered. The scattering data were recorded on a 1D complementary metal-oxide semiconductor (CMOS) Mythen detector (Dectris, Switzerland). Finally, the scattering data profile was collected as *I*(*s*) as a function of *s*, where *s* is momentum transfer vector defined as *s* = 4π(sinθ/λ) and units in 1/nm. The intensity profiles were collected for both CpGA and CpGB in water and PBS, and blank solvents from 5 to 50 °C at an interval of 5 to 10 °C. Table S1 summarizes all the programs used to process the acquired data. After the data collection, the beam position was set to zero using the software SAXSTreat, and SAXSQuant was used for buffer subtraction and desmearing the data to represent true point collimation. The SAXS data profiles of both the samples at different temperatures were analyzed to obtain radius of gyration (*R*_g_), maximum linear dimension (*D*_max_), and distance distribution function of pairwise vectors [*P*(*r*)] using the ATSAS 3.0.3 version. Using Guinier approximation for globular or rod-like scattering shape profiles provided *R*_g_ and radius of cross-section (*R*_c_), respectively. Persistence length of the molecules, *L*, and their aspect ratio, *A*, were estimated using *L* = (12((*R*_g_^2^ – *R*_c_^2^)))^1/2^ and *A* = *R*_g_/*R*_c_, respectively. Kratky analysis was also done using the same program. All the plots were made using ATSAS PrimusQT suite of programs. Shape restoration was done using ATSAS online server. Briefly, for all sets, 10 runs of DAMMIF program were done to model dummy residue uniform density models without any shape or symmetry bias. The solutions were aligned and averaged using offline version of DAMAVER suite of programs. Molecular maps were generated for the average and variable of the 10 dummy residue models. The images of the models were made using UCSF Chimera program version 1.13.

### Fluorescence correlation spectroscopy

The self-association of CpGA and CpGB was measured using FCS. FCS analyzes fluctuations in fluorescence intensity within the confocal volume to determine the diffusion times of fluorescent molecules present in nanomolar concentration. Alexa Fluor 488-labeled CpGA (20 nM) in the presence of unlabeled CpGA concentration ranging from 0 to 5 μM was used. Similarly, 20 nM Alexa Fluor 488-labeled CpGB in the presence of unlabeled CpGB concentration ranging from 0 to 5 μM was used. The FCS measurement was performed using an ISS Alba FFS/FLIM confocal system (Champaign, IL, USA), integrated with a Nikon Ti2U microscope (Nikon, Japan) equipped with the Nikon CFI PlanApo 60×/1.2 numerical aperture (NA) water immersion objective. The samples were drop casted on the 22-mm grease-free coverglass (Blue-Star, India). The samples were excited one at a time using a 488-nm picosecond pulsed diode laser, and FCS measurements were acquired. The fluorescence emission was detected using a SPAD (single-photon avalanche detector) detector and the 530/43-nm band-pass filter. The FCS correlation curves obtained were fit to the 3D Gaussian one-component diffusion model using VistaVision software (ISS Inc., USA). The fitted FCS correlation curves were plotted using OriginPro24 software (OriginLab, USA).

In the 3D Gaussian diffusion model, which considers a single type of diffusing molecule (the 3D Gaussian one-component model, excluding triplet state contributions), the correlation function *G*(τ) is defined by the following equation:

$$G\left(\tau \right)=\frac{1}{N\;}.{\left(1+\frac{\tau }{\tau_{D}}\right)}^{-1}.{\left(1+\frac{\tau }{S^{2}\tau_{D}}\right)}^{-\frac{1}{2}}\left[1+K_{\exp}\left(-\frac{\tau }{\tau_{S}}\right)\right]\;$$ (1)

where $\tau_{D}$ represents the diffusion time of the molecules, *N* is the average number of molecules within the observation volume, and *S* is the structural parameter that defines the ratio between the radius and the height of the confocal volume. The value of $\tau_{D}$, obtained by fitting the correlation function, is related to the diffusion coefficient (*D*) of a molecule through the following equation:

$$\tau_{D}=\frac{\omega^{2}}{4D}$$ (2)

where ω is the size of the observation volume. The beam waists in the radial and axial dimensions were calibrated using a standard fluorescence dye, Rhodamine 6G (R6G), in water with a known diffusion coefficient of 2.8 × 10^−6^ cm^2^/s. The fold change in the diffusion time was calculated with the mean diffusion time of 20 nM CpGA. The number of the particle (*N*) in the confocal volume was calculated using VistaVision software for the respective species.

### Isolation and culture of human pDCs

Peripheral blood samples were obtained from healthy adult donors after informed consent, the approval from institutional Human Ethics Committee at CSIR-Indian Institute of Chemical Biology, Kolkata, India, in accordance with the Declaration of Helsinki. Buffy coats were collected from the Blood Bank of Tata Medical Center, Kolkata, India, under an approved material transfer agreement. Blood was diluted 1:2 with PBS and layered over a Hisep LSM gradient for density centrifugation (2,500 rpm, 20 min, 25 °C, brake and acceleration settings = 1). The mononuclear cell layer was collected, washed, and subjected to hypotonic red blood cell lysis. Peripheral blood mononuclear cells (PBMCs) were resuspended in MACS buffer [PBS containing 0.5 mM EDTA and 0.5% bovine serum albumin (BSA)], and pDCs were isolated by magnetic-activated cell sorting (MACS) using anti-BDCA4-coated magnetic beads (Miltenyi Biotec) following Fc receptor blockade. Labeled cells were passed through LS columns on a magnetic stand, washed thoroughly, and eluted to obtain highly purified pDCs. Cells were cultured in complete RPMI 1640 medium in 96-well U-bottom plates at 37 °C with 5% CO₂ and processed as described in the figure legends.

### RNA interference in human pDCs

Primary human pDCs were subjected to siRNA-mediated knockdown using the 4D-Nucleofector System (Lonza) according to the manufacturer’s instructions. Briefly, cells were resuspended in 100 μl of supplemented P3 Primary Cell Solution and nucleofected with 175 ng of either a nontargeting enhanced green fluorescent protein (EGFP) control siRNA or a PIEZO1-specific siRNA or an ARHGAP26 siRNA using the program FF168. For gene knockdown, we used MISSION esiRNA from Sigma. These are not single siRNAs, but rather a heterogeneous mixture of siRNA that targets the same mRNA sequence. These multiple silencing triggers lead to highly specific and effective gene silencing (as mentioned by the manufacturer). Following nucleofection, cells were immediately transferred to RPMI 1640 medium supplemented with 10% fetal bovine serum and incubated at 37 °C in 5% CO₂ for 18 h prior to downstream analyses. Knockdown efficiency was validated by quantitative polymerase chain reaction (PCR) to assess *ARHGAP26* and *PIEZO1* mRNA levels.

### Flow cytometric analysis of CpG oligonucleotide uptake by human pDCs

CpGA and CpGB oligonucleotides were fluorescently labeled using either Alexa Fluor 647 or Alexa Fluor 488 dyes, following the protocol provided with the Ulysis Nucleic Acid Labeling Kit (Life Technologies). Primary human pDCs were preincubated for 20 min with Pitstop2 or Genistein before they were incubated with fluorescently tagged CpGA or CpGB for 30 min at 37 °C. Following incubation, cells were washed thoroughly with PBS to remove unbound material and immediately analyzed on a flow cytometer (BD LSRFortessa) to assess internalization based on fluorescence intensity.

### Scanning electron microscopy

Samples were prepared for scanning electron microscopy according to a previously published protocol [53]. Briefly, primary human pDCs were seeded onto poly-l-lysine-coated glass coverslips and allowed to adhere for 4 h at 37 °C. Cells were then stimulated with CpGA or CpGB for 10 min, followed by fixation in 2.5% glutaraldehyde at 4 °C overnight. After fixation, samples were washed 3 times with PBS (5 min each) to remove residual fixative, followed by 5 rinses with distilled water to eliminate salt residues. Cells were dehydrated through a graded ethanol series (30%, 50%, 70%, 90%, and 100%), and coverslips were air-dried completely at room temperature. Samples were imaged using an environmental scanning electron microscope (ESEM Quanta, Thermo Fisher Scientific).

### IRM: Global tension measurement

An inverted microscope (Nikon, Japan) with 60× 1.2 NA water immersion objective, Hg arc lamp, and necessary interference filter (546 ± 12 nm) were used for the IRM [30,54]. IRM imaging of pDCs was achieved by using magnetic beads and magnet under the coverslip to bring them closer to the glass substrate and enhance interaction with the substrate. Cells that were adhered were used for imaging. Fast time-lapse images at an interval of 20 ms, with 50 frames per second frame rate, were captured using a sCMOS (scientific complementary metal-oxide semiconductor) camera. Over 8,192 frames were recorded to capture the fast transient changes in mechanical properties caused by cargo oligonucleotide addition (Hamamatsu, Japan).

### TIRF microscopy coupled with IRM microscopy: Local tension measurement

The cells were kept on the microscope stage top incubator (Tokai Hit, Japan) for 10 min for the system to stabilize. For correlative TIRF-IRM sequential imaging, Eclipse Ti-E motorized inverted microscope (Nikon, Tokyo, Japan) equipped with 100× 1.4 NA water immersion objective and sCMOS (ORCA Flash 4.0 Hamamatsu, Japan) was used. For IRM-based imaging, a 100-W Hg arc lamp with necessary interference filters (546 ± 12 nm) and 50:50 beam splitter was used [30,55,56]. For TIRF imaging, coherent OBIS laser of 488 nm was used [57–59].

Human pDCs cells were seeded on poly-d-lysine-coated confocal dishes and were incubated at 37 °C, 5% CO_2_ for 45 min for them to adhere to the glass substrate. After 45 min of cell seeding, the medium was replenished and cells were taken for imaging. Properly adhered cells were chosen for imaging. Calibration for membrane fluctuation tension mapping was done using 60-μm-diameter polystyrene NIST (National Institute of Standards and Technology) beads (Bangs Laboratories Inc.). The sequence followed for correlative TIRF-IRM sequential imaging for any field that had fluorescent signal was TIRF-IRM-TIRF. For time points before oligonucleotide addition (no fluorescence), only IRM frames were captured. For IRM imaging, time-lapse images at an interval of 50 ms with 20 frames per second rate were captured. For a particular field 2,048 frames were captured for effective membrane fluctuation tension measurement. This first IRM movie was considered as the baseline without the oligonucleotide addition. Fluorescein isothiocyanate (FITC)-tagged CpGA (1 μM) was subsequently added to the medium without disturbing the field of view. Following the same field at 5, 15, and 20 min of addition of oligonucleotides, sequential TIRF-IRM images were captured. For TIRF imaging, images were acquired at an interval of 200 ms for 300 of the same field as that of IRM at a frame rate of 3.33 frames per second with a penetration depth of approximately 90 to 100 nm. TIRF movies captured before and after the IRM movie were used to verify the stability of the observed fluorescent pattern over the 1- to 2-min imaging period. Same protocol as followed in the case of CpGA was used to determine the effect of CpGB on local tension profiles of the plasma membrane.

### Analysis of IRM data

Proper adherence of the cells to the glass substrate was ensured for selecting them for analysis. Further, from these selected adhered cells, only regions that were within a range of ~100 nm from the coverslip—also called the first branch region (FBR)—were chosen and the size of these chosen FBRs was 4 × 4 pixels, corresponding to ~65 nm × 65 nm per pixel for IRM images of the TIRF-IRM sets and ~(300 nm)^2^ for only IRM images. To maintain stringency in selecting the subset of regions for extracting parameters and estimating the membrane height fluctuations, the methodology designed previously [30,55] has been followed.

For analyzing the mechanical parameters of the cells under these 2 conditions over the time points, the PSD was estimated by autoregression technique [probability of covariance (pcov)] (MATLAB, Mathworks Inc., USA). For estimating active temperature (*A*), effective cytoplasmic viscosity (η_eff_), confinement (γ), and membrane tension (σ), PSD is fitted to the Helfrich-based theoretical model [26]:

$$\mathrm{PSD}\left(f\right)=\frac{4\eta_{\mathrm{eff}}Ak_{B}T}{\pi }\int_{q_{\min}}^{q_{\max}}\frac{\mathrm{dq}}{{\left(4\eta_{\mathrm{eff}}\left(2\pi f\right)\right)}^{2}+{\left[\kappa q^{3}+\sigma q+\frac{\gamma }{q}\right]}^{2}}$$ (3)

The fitting was done using MATLAB, and fits with *R*^2^ > 0.9 were considered [55]. The standard deviation of the membrane height over time series of a single pixel is estimated and averaged over single FBRs to obtain the membrane height fluctuations termed as SD_time_. Generally, effective membrane tension is termed as the fluctuation tension as the interpretation is dependent on the framework of membrane fluctuations [31]. The effective membrane fluctuation tension is termed as “tension” in the text. It has contributions from all players affecting membrane fluctuations, including the cytoskeleton.

For tension mapping (Fig. 1G), PSDs calculated from each pixel were fitted and all parameters were extracted from fits—including tension and *R*^2^. The pixel-wise standard deviation of the membrane height (SD_time_) over time series was also mapped.

### TIRF-IRM correlative analysis

The first frame of the TIRF movie obtained after the IRM movie was used to obtain the pixel-wise correlation of local intensity with pixel-wise-fitted tension and pixel-wise SD_time_ at the sites where FITC-tagged oligonucleotide binds to the plasma membrane. Template matching was used to align frames of IRM movies to remove any effect of stage drift and to ensure proper alignment between IRM and fluorescence fields. This was done for every cell and for each time point. At the peak of fluorescence of each cluster in cells in the first frame of the TIRF movie obtained after the IRM movie, line scans (11 pixels along the *x* direction and 11 pixels along the *y* direction) are performed for 10 to 20 clusters of CpGA per time point. These line scans were mapped onto pixel-wise tension and pixel-wise SD_time_. This correlative mapping was carried out for the baseline (no treatment) as well as 5, 15, and 20 min after addition of oligonucleotides. For all the time points, the first frame of TIRF movie captured after its corresponding IRM movie was used for this correlative mapping. For plotting the baseline condition, the regions where clusters appeared on the first frame of the first TIRF movie captured after 5 min of CpGA addition were used to trace back the basal local membrane height fluctuation and tension in the untreated condition at those local sites where the oligonucleotide binds after addition. The center is normalized to one for all scans of CpGA/CpGB clusters followed by averaging over 10 to 20 clusters per time point. The same cells were followed in time, but puncta were independently chosen for each time point. Error is denoted as SEM. The profiles were also averaged based on distance from center. Only for better representation purposes, both negative and positive distance were plotted; however, the data are the same for 2 points at the same distance from the center. CpGA/CpGB puncta very close to cell edge were not taken into account to avoid arbitrary tension values expected for pixels lying outside the cell. During the correlation mapping at the clusters, pixels where PSD fitted to *R*^2^ > 0.9, membrane fluctuation tension < 5,000 pN/μm and SD_time_ < 15 nm were only considered. Mann–Whitney *U* test was performed to calculate the statistical significance of the various mechanical properties at various time points with respect to the baseline. From the center of the cluster, the increase in tension within 325 nm was calculated and the probability distribution of maximum surge in tension was plotted for all the conditions. Also, the mean pixel-wise local tension for 10 × 10 pixels around the center of the cluster, normalized with respect to baseline condition, was also calculated and plotted.

### Immunostaining for confocal imaging

Primary human pDCs were seeded onto poly-d-lysine-coated (1 μg/ml) coverslips at a seeding density of 0.05 × 10^6^ cells per coverslip and incubated at 37 °C in 5% CO_2_ for 2 to 3 h for attachment. For PIEZO1 localization studies, cells were either left untreated (control) or stimulated with either CpGA (250 nm) or CpGB (250 nm). After incubation, cells were fixed in 4% paraformaldehyde for 15 min at room temperature. Following fixation, cells were blocked in 3% BSA in PBS containing 0.2% Triton X-100 for 60 min at room temperature. Cells were incubated overnight at 4 °C with rabbit anti-human Piezo1 antibody (Proteintech) at 1:100 dilution (5 μg/ml). The next day, Alexa Fluor 568-conjugated anti-rabbit immunoglobulin G (IgG) (1:200 dilution) was applied for 60 min at room temperature. After washing, coverslips were mounted with Vectashield containing 4′,6-diamidino-2-phenylindole (DAPI) for nuclear staining and sealed for confocal imaging.

For endosomal colocalization analysis, pDCs were pretreated with GsMTx4 (10 μM) for 30 min, followed by addition of FITC-tagged CpGA for 15 min. Cells were washed with PBS to remove unbound CpGA and seeded onto precoated poly-d-lysine (1 μg/ml)-coated coverslips for adherence. After 15 min, cells were fixed and blocked as described above. Immunostaining was performed using mouse anti-human EEA1 (eBioscience) and rat anti-human LAMP1 (Abcam), both at dilution of 1:100 (5 μg/ml), followed by overnight incubation at 4 °C. Alexa Fluor 568-conjugated anti-mouse IgG and Alexa Fluor 647-conjugated anti-rat IgG secondary antibodies were applied at 1:200 dilution for 60 min. Cells were mounted with Vectashield containing DAPI prior to confocal imaging.

For assessment of transcription factor nuclear translocation, primary human pDCs were stimulated for 30 min with 2 μM CpGA in the presence or absence of GsMTx4 (10 μM) or stimulated with CpGB in the presence or absence of Yoda1 (15 μM). Cells were fixed, permeabilized, and blocked as described above. Immunostaining was performed using mouse anti-human phospho-IRF7 (Ray Biotech 128-10356-3) and rabbit anti-human NF-κB–p65 antibodies (both at 1:100 dilution). Subsequent detection was performed using goat anti-rabbit Alexa Fluor 568 and goat anti-mouse Alexa Fluor 488 antibodies (both at 1:200 dilution). Nuclei were counterstained with Hoechst dye. For quantification of nuclear versus cytoplasmic fluorescence intensity density, regions of interest (ROIs) were generated in ImageJ/Fiji as follows: Nuclei were segmented using the DAPI channel—to create a clean nuclear mask (ROI_n_). The full cell boundary was delineated using bright-field images, with manual ROI tracing to produce a whole-cell ROI (ROI_x_). The cytoplasmic ROI (ROI_c_) was then defined by subtracting the nuclear mask from the whole-cell ROI (ROI_c_ = ROI_x_ – ROI_n_). Fluorescence intensity density of NF-κB and IRF7 was measured separately within ROI_n_ (nuclear) and ROI_c_ (cytoplasmic) regions. Finally, the nuclear-to-cytoplasmic intensity ratio was calculated by dividing the nuclear intensity density by the cytoplasmic intensity density.

For endosomal and actin colocalization studies, the protocol for endosomal staining was followed. For actin visualization, primary human pDCs were stained with Alexa Fluor 532-tagged phalloidin (1:200 dilution; 0.5 μg/ml) following CpGA addition in the presence or absence of GsMTx4.

### Confocal microscopy image analysis

For counting the fraction of compartment endosomal or puncta that have spatial codistribution with actin puncta (in MATLAB), a mask was applied over the image to select the cell. For this cell, the equatorial z slices were selected, and thresholding was performed using the appropriate threshold, resulting in a binary image for both the endosomal and actin signal. Single pixels were removed using serial erosion and dilation, and subsequently, the binary image was used to detect objects. It was observed that the actin objects from the cortex were detected as a single large continuous object. For selecting actin objects away from the cortex, an object-size-based threshold was applied in addition to the usual procedure of applying local intensity thresholding with a proper sensitivity factor. Objects were detected for each channel separately. The pixels overlapping in the 2 binary images were considered to be colocalizing, and another binary image/matrix for the colocalized pixels was constructed. Colocalized objects were detected from this newly formed matrix using 8-pt connectivity (MATLAB) object detection. The sum of the number of all the colocalized objects in all the z slices divided by the total number of endosomal objects detected in all z slices gave us the fraction of objects that overlapped with actin per cell. Mann–Whitney *U* test was used to calculate significance, unless specified (ns denotes *P* > 0.05, *P* < 0.05 is denoted as *, and *P* < 0.001 is denoted as ** statistical significance).

### ELISA for IFN-α and TNF-α

Concentration of IFN-α and TNF-α in pDC culture supernatants was determined using sandwich enzyme-linked immunosorbent assay (ELISA) (Mabtech, Sweden) according to the manufacturer’s protocol.

### RNA isolation and real-time PCR

Total RNA was extracted using TRIzol reagent (Invitrogen), following the manufacturer’s protocol. RNA was reverse-transcribed into complementary DNA (cDNA) using the SuperScript III First-Strand Synthesis System (Invitrogen). Quantitative real-time PCR was performed on an Applied Biosystems 7500 Fast Real-Time PCR System using SYBR Green chemistry. To measure the relative expression of genes, we performed quantitative real-time PCR. For each sample, CT (cycle threshold) values were obtained for both the target gene and the housekeeping gene, and technical triplicates were included for each condition. The average CT for the housekeeping gene was calculated for each sample set and used to normalize the target gene expression. Specifically, we calculated ΔCT by subtracting the average housekeeping gene CT from the target gene CT (ΔCT = CT_target − CT_housekeeping). To quantify relative expression, we used the formula (2^−ΔCT^) × 100,000, where the constant scaling factor (100,000) was used. The relative expression values obtained from each technical replicate were then averaged to generate a representative expression value for each experimental condition. Primer sequences used for gene expression analysis are provided in Table S2.

### Flow cytometry for calcium influx

Isolated primary human pDCs were stained with the calcium-sensitive fluorescent dye Fluo-3 AM (1.5 μM; Invitrogen) for 30 min at 37 °C in PBS supplemented with 1.2 mM CaCl₂ and 2% fetal bovine serum (GIBCO). After incubation, cells were washed twice in the same buffer and incubated at room temperature for an additional 30 min to allow complete de-esterification and efflux of unincorporated dye. Stained cells were then acquired on a BD LSRFortessa flow cytometer at the indicated time points before and after the addition of specific treatments. Changes in intracellular calcium levels were monitored by measuring the mean fluorescence intensity (MFI) in the FITC channel (corresponding to Fluo-3 AM), with increased MFI reflecting calcium mobilization in response to stimulation.

### Subcellular fractionation

Subcellular fractionation was performed according to a previously published protocol [60]. Primary human pDCs were incubated with biotinylated CpGB (CpGB-biotin, 5 μM) in the presence or absence of Yoda1 (10 μM) for 30 min at 37 °C. After stimulation, cells were immediately transferred to ice, washed with cold PBS, and processed for subcellular fractionation using an iodixanol gradient. Briefly, 2 × 10^6^ to 3 × 10^6^ cells per condition were resuspended in hypotonic lysis buffer containing 50 mM Hepes (pH 7.8), 78 mM KCl, 4 mM MgCl₂, 8.4 mM CaCl₂, 10 mM EGTA, 250 mM sucrose, 100 μg/ml cycloheximide, 5 mM vanadyl ribonucleoside complex, and an EDTA-free protease inhibitor cocktail (Roche). Cells were allowed to swell on ice for 10 min and lysed by 20 to 25 strokes with a glass Dounce homogenizer (Sartorius). The homogenate was centrifuged at 1,000*g* for 10 min at 4 °C to remove unbroken cells and nuclei. The post-nuclear supernatant was layered onto a preformed discontinuous iodixanol gradient (3% to 30%, prepared in gradient buffer) and ultracentrifuged at 36,000 rpm for 5 h at 4 °C in an SW60 Ti rotor (Beckman Coulter). Fractions were manually collected from the top in equal volumes and maintained on ice. Endosome-enriched fractions were identified based on density and lysed by adding saponin to a final concentration of 0.1% (w/v), followed by incubation on ice for 20 min. To quantify the distribution of CpGB-biotin across fractions, 200 μl of fractions was incubated overnight at 4 °C in high-binding 96-well ELISA plates (Nunc). After washing with PBS containing 0.05% Tween 20 (PBST), wells were incubated with streptavidin–horseradish peroxidase (HRP; 1:5,000) for 2 h at room temperature. Plates were washed again with PBST and developed using the TMB (3,3′,5,5′-tetramethylbenzidine) substrate, and the reaction was stopped with 1 N HCl. Absorbance was measured at 450 nm using a microplate reader. Optical density values were used to assess subcellular distribution of CpGB under different treatment conditions.

### Statistics

Data were plotted and analyzed using GraphPad Prism v8.4.2. Statistical significance was assessed using unpaired Student’s *t* tests, unless otherwise specified in the figure legends. A *P* value of <0.05 was considered statistically significant. The number of independent replicates for each experiment is indicated in the corresponding figure legends.

## Data Availability

Data will be available on request.
